# Pediatric Influenza-Associated Deaths in New York State: Death Certificate Coding and Comparison to Laboratory-Confirmed Deaths

**DOI:** 10.1155/2012/397890

**Published:** 2012-09-25

**Authors:** Dina Hoefer, Bryan Cherry, Marilyn Kacica, Kristi McClamroch, Kimberly Kilby

**Affiliations:** ^1^Bureau of Communicable Disease Control, New York State Department of Health, Corning Tower Room 651, Albany, Empire State Plaza, NY 12237, USA; ^2^Department of Epidemiology and Biostatistics, University at Albany School of Public Health, Rensselaer, NY 12144, USA; ^3^Department of Family Medicine, Albany Medical College, 47 New Scotland Avenue, MC-34, Albany, NY 12208, USA

## Abstract

*Introduction*. Surveillance for laboratory-confirmed influenza-associated deaths in children is used to monitor the severity of influenza at the population level and to inform influenza prevention and control policies. The goal of this study was to better estimate pediatric influenza mortality in New York state (NYS). *Methods*. Death certificate data were requested for all passively reported deaths and any pneumonia and influenza (P&I) coded pediatric deaths occurring between October 2004 and April 2010, excluding New York City (NYC) residents. A matching algorithm and capture-recapture analysis were used to estimate the total number of influenza-associated deaths among NYS children. *Results*. Thirty-four laboratory-confirmed influenza-associated pediatric deaths were reported and 67 death certificates had a P&I coded death; 16 deaths matched. No laboratory-confirmed influenza-associated death had a pneumonia code and no pneumonia coded deaths had laboratory evidence of influenza infection in their medical record. The capture-recapture analysis estimated between 38 and 126 influenza-associated pediatric deaths occurred in NYS during the study period. *Conclusion*. Passive surveillance for influenza-associated deaths continues to be the gold standard methodology for characterizing influenza mortality in children. Review of death certificates can complement but not replace passive reporting, by providing better estimates and detecting any missed laboratory-confirmed deaths.

## 1. Introduction

It has long been recognized that influenza is associated with substantial mortality during both epidemics and pandemics. Death due to influenza virus infection can result from a variety of causes, such as pneumonia or exacerbations of existing cardiopulmonary or other chronic conditions. Influenza-associated death among children in particular is rare, but when it occurs, it is often rapidly fatal and may affect children with no predisposing risk factor [1, 2]. Bacterial coinfections, especially methicillin-resistant *Staphylococcus aureus *(MRSA), are also increasingly being documented among influenza-associated pediatric deaths [1]. These were important factors in the Advisory Committee on Immunization Practices (ACIP) expanding influenza vaccine recommendations in 2008 to include all children aged 6 months through 18 years [3] when in years prior influenza vaccine was only recommended for children less than five years of age. 

Due to increased reports of deaths in children associated with influenza in the 2003-04 season [4, 5], in October 2004 laboratory-confirmed influenza-associated deaths in children (<18 years of age) became a nationally notifiable condition to the United States (US) National Notifiable Diseases Surveillance System (NNDSS). Goals of the national surveillance system are (1) to monitor and describe the incidence, distribution, and basic epidemiologic characteristics of deaths among children related to infection with influenza, (2) provide data to guide future influenza immunization policy, and (3) recognize influenza seasons in which the impact of influenza is unusually severe among children [5]. This is a passive surveillance system with known limitations [4, 6, 7].

In addition to NNDSS reporting of pediatric deaths, national mortality data of the entire population has been used extensively to estimate influenza-associated deaths in the US over time. These estimates vary depending on the methods used [8] and are important for informing influenza prevention and control strategies. It is common practice to combine pneumonia and influenza deaths apply statistical methods to generate estimates because pneumonia mortality correlates closely with influenza mortality [9]. In New York state (NYS), six cities outside of New York City report data to the 122 Cities Mortality Reporting System, the Centers for Disease Control and Prevention (CDC) surveillance for pneumonia and influenza (P&I) deaths [10]. In NYS, statewide P&I data is not utilized to monitor death during the influenza season due to inherent delays in reporting of vital statistics data. 

Surveillance for laboratory-confirmed pediatric influenza-associated deaths and P&I surveillance are unlikely to be comprehensive and may underestimate deaths due to influenza in children. To date, no studies have systematically compared laboratory-confirmed influenza-associated deaths in children to mortality data as reported on death certificates. The goals of the current study were to (1) compare influenza-associated pediatric deaths reported to the New York state Department of Health (NYSDOH) to all pediatric deaths reported on NYS death certificates as attributable to pneumonia and influenza, and (2) produce a better estimate of the total number of deaths due to influenza in NYS children that occurred between October 2004 and April 2010. Comparing two distinct surveillance systems is a quick and inexpensive method to more closely estimate a true mortality burden [11]. The results of this comparison will be useful for assessing the representativeness of these surveillance methods and to inform and improve existing methodologies and influenza surveillance efforts in NYS. 

## 2. Methods

Deaths among pediatric patients suspected or confirmed to be related to any type of influenza became a reportable condition, mandated under the NYS Sanitary Code (10NYCRR 2.10.2.11) in December 2004. Influenza-associated deaths in children are reported by clinicians, laboratories, and medical examiners to the local health department where the case resides (regardless if death occurred elsewhere) which in turn report the death to NYSDOH. A confirmed influenza-associated pediatric death is defined as a death in a child aged less than 18 years with clinically compatible illness and positive laboratory evidence of influenza on pre- or postmortem clinical specimens utilizing rapid influenza diagnostic tests, viral isolation, enzyme immunoassay, immunofluorescent antibody staining, immunohistochemical staining, or reverse-transcriptase polymerase chain reaction (RT-PCR) testing. 

All laboratory-confirmed pediatric influenza-associated deaths reported to the NYSDOH from October 2004 through April 2010, excluding New York City (NYC) residents, were utilized for this paper. A national standard case report form was used to collect information on patient demographics, time/place of death, influenza and invasive bacterial pathogen testing results, underlying medical conditions, medical care received, clinical diagnoses, and complications, and medication and influenza vaccination history. In NYS, local health department staff and NYSDOH epidemiologists work together to complete the case report form for each confirmed pediatric death. 

NYS death certificate data were requested from NYSDOH Bureau of Biometrics and Health Statistics for all reported laboratory-confirmed pediatric influenza-associated deaths. Death certificates were also obtained for all pediatric deaths occurring between October 2004 and April 2010, excluding NYC, where pneumonia and/or influenza were coded as the underlying cause of death (Table 1). Data extraction was based on the underlying cause of death, as it historically represents the disease or injury that initiated the chain of morbid events that led directly to the death [12]. Underlying cause of death is categorized using the International Classification of Diseases, 10th Revision (ICD-10). In NYS, a physician reports the immediate cause, up to two “due to or the consequence of” conditions, and “other significant conditions contributing to death but not related to the cause given as the immediate cause.” The reported cause and conditions of death are reviewed automatically using software provided by the National Center for Health Statistics (NCHS): ICD-10 ACME Decision Tables for Classifying Underlying Cause of Death. The software system uses the information to assign an underlying cause of death, which is reported in the NYSDOH Death File. In cases where the software cannot assign a cause, a cause is assigned manually by NYSDOH Bureau of Production Systems.

Laboratory-confirmed pediatric influenza-associated deaths were matched with P&I coded deaths by name, date of birth, and date of death to determine which deaths were identified by both surveillance systems. For all pneumonia coded deaths that did not match a laboratory-confirmed pediatric influenza-associated death record the hospital medical record was requested for review, when available, to determine if influenza testing was done to rule out influenza as a possible cause or contributor to illness. Univariate frequencies were calculated, stratifying by surveillance system when applicable. 

The total number of influenza-associated deaths among children in NYS was estimated using Petersen's capture-recapture method [11, 13]. Peterson's estimator, *N* (total deaths), was calculated by equating the observed rates from both surveillance systems which gives *N* = (*n*_1_ × *n*_2_)/*m*_2_. The first surveillance system laboratory-confirmed influenza-associated deaths captured *n*_1_ cases giving a capture rate of *n*_1_/*N*. The second system P&I surveillance captured *n*_2_ cases, including *m*_2_ cases that were already captured by the first system (recaptured or matched deaths). The recapture rate was *m*_2_/*n*_2_. Peterson's estimate implies that the estimated number of cases missed by both systems (*z*) equals (*b* × *c*)/(*a*); where *b* is the number of captured deaths by P&I surveillance only, *c* is the number of deaths captured by laboratory-confirmed influenza-associated pediatric death surveillance, and *a* represents the number of matched deaths (*m*_2_) (Table 2). The final estimated number of deaths was calculated by adding *a* + *b* + *c* + *z*. This estimate is valid under the assumption that the probability of being captured by one system does not affect the probability of being captured by the other, that the study population remained approximately constant without significant migration during the study period, and that ascertainment of meeting case definition by both surveillance systems was valid [11, 13]. All analyses were performed with SAS software, version 9.1.

## 3. Results

 Between October 2004 and April 2010, 34 laboratory-confirmed influenza-associated pediatric deaths have been reported to the NYSDOH. More than half of the cases (19/34, 55.8%) were infected with the 2009 pandemic influenza A H1N1 (pH1N1) virus. Excluding the unknown, most children were treated in the intensive care unit (14/27, 51.9%) and had a preexisting high-risk medical condition (17/32, 53.1%). The median time from symptom onset to death was four days.

### 3.1. Matching Analysis

**Figure 1:**
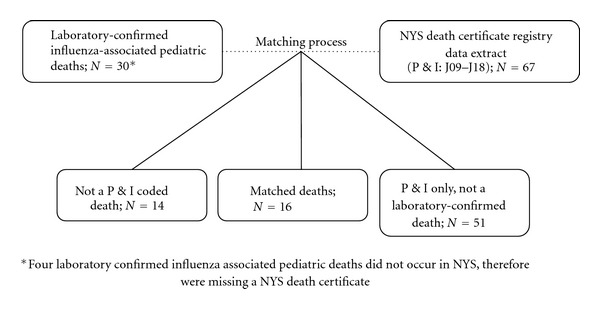


**Figure 2:**
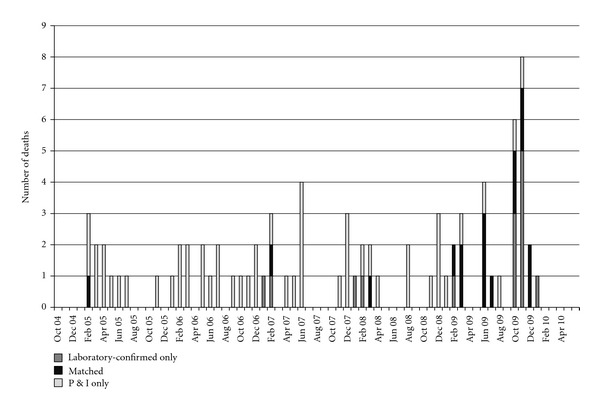
Influenza and pneumonia pediatric deaths by month and year.

Among the 34 laboratory-confirmed influenza-associated pediatric deaths, 4 did not have an NYS death certificate because the death occurred outside of NYS; these were excluded from all further analysis. A P&I code was noted on 67 death certificates among NYS children between October 2004 and April 2010. Among the 30 laboratory-confirmed influenza-associated pediatric deaths with an NYS death certificate, 16 matched a P&I coded death (16/30, 53.3%) (Figure 1). The combined laboratory-confirmed influenza-associated pediatric death and unmatched P&I coded pediatric deaths over time totaled 81 deaths. In total, most deaths occurred during the months of October to December (29/81, 35.8%), followed by January to March (26/81, 32.1%), and April to June (18/81, 22.2%) (Figure 2). Few deaths occurred during the summer months of July to September (8/81, 9.9%). Prior to the onset of the 2009 H1N1 pandemic in April 2009 [14], most deaths occurred in January to March (25/58, 43.1%). After the pandemic onset, deaths were predominantly laboratory-confirmed influenza-associated pediatric deaths, with only four additional deaths identified through P&I only surveillance during this timeframe. 

The majority of matched (13/16, 81.2%) and unmatched (11/14, 78.6%) laboratory-confirmed influenza-associated pediatric deaths were greater than 5 years of age while the majority of P&I only surveillance deaths were under the age of 5 years (39/51, 76.5%) (Table 3). The majority of matched (9/16, 56.3%) and unmatched (9/14, 64.3%) laboratory-confirmed influenza-associated pediatric deaths were confirmed pH1N1 influenza infections. When an autopsy was performed, the findings were used to determine the underlying cause of death more frequently for matched (10/11, 90.9%) and unmatched (10/10, 100%) laboratory-confirmed influenza deaths than P&I only surveillance deaths (27/38, 71.1%). 

### 3.2. Laboratory-Confirmed Influenza-Associated Deaths

Among the 16 laboratory-confirmed influenza-associated pediatric deaths that matched a P&I death certificate, all had an underlying cause of death coded as influenza (ICD-10 codes J09–J11). Among the 14 laboratory-confirmed influenza-associated pediatric deaths that did not match a P&I death certificate, the most common underlying cause of death coded on 3 death certificates (3/14, 21.4%) was “Other ill-defined & unspecified cause”. All three of these deaths occurred during the 2009-2010 influenza season. The remaining 11 unmatched deaths each had a different code for underlying cause of death listed on the death certificate. When all ICD-10 codes were reviewed beyond the underlying cause of death, two additional death certificates contained an influenza ICD-10 code, one contained a pneumonia ICD-10 code, and one record contained both influenza and a pneumonia ICD-10 code. 

### 3.3. Pediatric P&I Surveillance Deaths

Among the 51 P&I only surveillance deaths, only four had an underlying cause of death coded as influenza (J09–J11); the majority (47/51, 92.2%) were pneumonia coded deaths (J12–J18). One influenza coded death had a known commercial influenza positive laboratory result for influenza B virus and would have met the national case definition for reporting to the NYSDOH. Among the 47 pneumonia coded deaths, 7 (14.9%) were viral pneumonia, 9 (19.1%) were bacterial pneumonia, and 31 (66.0%) were pneumonia, organism unspecified. When all of the codes on each death certificate were reviewed for the 47 pneumonia coded deaths, no records contained an influenza ICD-10 code. Medical records were requested for 34/47 (72.3%) pneumonia coded deaths, as these deaths occurred in the hospital. A record was available for review for 33 out of the 34 (97.1%). Among the reviewed records, 7/33 (21.2%) had documented influenza testing and of those 7 (100%) tested negative.

### 3.4. Capture-Recapture Analysis

The capture-recapture analysis (Table 4) comparing the 30 deaths detected by laboratory-confirmed influenza-associated pediatric death surveillance and the 67 P&I coded deaths estimated that there were 45 undetected influenza-associated deaths for an estimated number of 126 influenza-associated deaths in children between October 2004 and May 2010. When this analysis was limited to comparing the 30 deaths detected by laboratory-confirmed influenza-associated pediatric death surveillance and only influenza coded deaths certificates, of which there were 20, only 4 influenza-associated deaths were undetected for an estimated number of 38 influenza-associated deaths in children.

## 4. Discussion

 After six years of conducting surveillance for laboratory-confirmed influenza-associated deaths among children in NYS, only a very small number of deaths have been reported. While rare, it is important to continue to monitor these deaths and improve vaccination coverage among children in order to prevent influenza and its complications. It is recognized that reported deaths likely underestimate the direct impact of influenza as a cause of death among children because of an overall low baseline level of testing children for influenza, poor sensitivity and low positive predictive value of rapid influenza tests, and underreporting of known cases due to passive nature of surveillance [6, 15]. Based on the analysis reported here, estimation of influenza-associated mortality in children is similarly underestimated when based on cause of death data reported on death certificates. Many death certificates have no mention of influenza as an immediate or contributing cause of death, even among children with laboratory-confirmed influenza infection. Determining the most appropriate death category for characterizing the burden of influenza mortality is difficult and debated [9, 16–18].

 In studies, when influenza mortality is analyzed based on death certificate data, pneumonia coded deaths are often combined with influenza coded deaths [19]. Not all pneumonia is caused by influenza, but it is widely agreed that many influenza deaths are being coded as pneumonia deaths [20]. This is due to recognized spikes in pneumonia mortality that correlate with influenza seasons [21]. However, the accuracy of this assumption for children has not been studied, despite the data being used to assess influenza disease severity among children [19]. 

In this study the P&I pediatric deaths included were much younger than known laboratory-confirmed influenza-associated deaths. None of the laboratory-confirmed influenza-associated pediatric deaths reported to the NYSDOH had a pneumonia ICD-10 code as the underlying cause of death. Also, when hospital records for pneumonia coded deaths were reviewed, influenza testing was ordered in less than a quarter of these deaths and found to be negative each time. Taken together, this evidence suggests there are distinct differences in the population of children coded as dying from pneumonia on their death certificates and those who die and have laboratory evidence of influenza. There may be differences in the risk factors, testing practices for influenza, or coding practices by age. Past research has also noted primary influenza pneumonia occurs most commonly in adults and may progress rapidly to acute lung injury requiring mechanical ventilation [22]. Unlike in adults, influenza pneumonia in children is usually a benign illness, and the mortality is low [23]. Secondary bacterial infection seems to be a more common complication of influenza infection in children [1, 23, 24]. 

Death certificate coding practices can highly influence mortality data trends. It has been found that during past influenza pandemics physicians and medical examiners were more likely to code deaths clinically compatible with pneumonia and influenza as being caused by influenza [9]. Most likely, the knowledge that a pandemic was underway resulted in diagnostic substitution of an influenza-specific ICD code for the nonspecific ICD pneumonia codes used more commonly during nonpandemic years [9]. Our analysis found similar findings during the 2009 H1N1 pandemic when a shift occurred from most deaths being detected by P&I surveillance to most deaths being detected by laboratory-confirmed influenza-associated pediatric death surveillance. Only four additional pneumonia coded deaths were found during the pandemic period. This is not likely due to an actual decrease in pneumonia deaths. Autopsy may also play an important role in coding practices as influenza-associated deaths can be detected after the child expires with post-mortem testing. It is important then to ensure that follow-up amendment of death certificates codes it done if an accurate picture of influenza as an underlying cause of death is to be captured. Overall, the use of correct codes when completing death certificates and educating clinical and medical examiners on the importance coding is critical for the utility of ICD-10 code-based surveillance and research of influenza-associated deaths in children. 

Among the 30 laboratory-confirmed influenza-associated pediatric deaths, no single ICD-10 code besides influenza codes was used consistently. Similar analyses could be conducted in other states, matching their laboratory-confirmed influenza-associated pediatric deaths to death certificate data, to further review underlying cause of death ICD-10 coding. The compiling of such data into a larger and more geographically diverse sample would make the results more generalizable and may lead to better mortality estimates of influenza-associated deaths in children. Annual matching of laboratory-confirmed influenza-associated pediatric deaths to death certificates and review of any additional influenza ICD-10 coded deaths that were not reported can be done with minimal effort and be used to find missed deaths that would have met CDC case definition. This activity will be continued in NYS.

The capture-recapture analysis estimated that between 38 and 126 influenza-associated pediatric deaths occurred in NYS since the 2004-2005 influenza season through the 2009-2010 season. If the lower estimate is accurate, then very few influenza deaths are being missed by the passive surveillance methods for laboratory-confirmed influenza-associated pediatric deaths in NYS. The higher bound, which includes pneumonia coded deaths, may be a better estimate if there weas more confidence that pneumonia coded deaths accurately reflect influenza in children. It is likely that the true influenza-related mortality burden in NYS falls somewhere in the middle. The analysis presented here suggests that P&I surveillance methods may not be applicable to estimation of influenza-related mortality among children, but this would require further study. 

A limitation that was known prior to beginning this analysis was that pediatric influenza-associated deaths are passively reported to NYSDOH. Passive reporting of pediatric influenza-associated deaths is known to be subject to underreporting and variations in data collection from season to season [25]. They also require laboratory confirmation as part of the case definition. Testing practices are based on the point-of-care process and can also vary from season to season [15], especially when media coverage heightens awareness of influenza deaths among children. While NYC and NYS deaths are aggregated and reported together publicly during the influenza season, NYC data was not included in this analysis as the NYC Department of Health and Mental Hygiene (NYCDOHMH) reports their own influenza-associated pediatric death case report data directly to CDC. Finally, the number of pediatric influenza-associated deaths was small and limited analyses and conclusions that could be drawn. 

## 5. Conclusion

Modeling estimates from the CDC and several other groups build on decades of work devoted to better understanding of how to use US vital statistics data to estimate the burden of influenza for the whole population [8]. These estimates in turn are important for informing influenza prevention and control policies. For children alone, passive, case-based surveillance for influenza-associated deaths continues to be the gold standard methodology for characterizing influenza mortality in children despite its limitations. Findings from national analyses of reported influenza-associated pediatric deaths emphasize the need to improve vaccination coverage among all children, especially those at increased risk for influenza-related complications [26]. Annual review of pediatric death certificate data can complement passive reporting, by providing better estimates and detecting any missed laboratory-confirmed deaths, but it cannot replace case-based surveillance. 

## Figures and Tables

**Table 1:** 

ICD-10 code	Description
J09*	Influenza due to identified avian influenza virus
J10	Influenza due to other identified influenza virus
J11	Influenza, virus not identified
J12	Viral pneumonia, not elsewhere classified
J13	Pneumonia due to *Streptococcus pneumonia *
J14	Pneumonia due to *Haemophilus influenza *
J15	Bacterial pneumonia, not elsewhere classified
J16	Pneumonia due to other infectious organisms, not elsewhere classified
J17	Pneumonia in diseases classified elsewhere
J18	Pneumonia, organism unspecified

*This code was used to designated pH1N1 related deaths.

**Table 2:** Capture-recapture analysis using two independent surveillance systems.

		Laboratory-confirmed influenza	
		pediatric death surveillance	
		Reported	Missed

P&I surveillance	Reported	*a* (*m*_2_)	*b*
	Missed	*c*	*z*

*n*_1_ = *a* + *c*.

*n*_2_ = *a* + *b*.

*N* = *a* + *b* + *c* + *z*.

Peterson's estimateor of *N* (total deaths): *N* = (*n*_1_ × *n*_2_)/*m*_2_  [13].

**Table 3:** Demographic characteristics and underlying cause of death.

	Laboratory-confirmed pediatric		Matched deaths		P&I surveillance only	
	Influenza-associated death surveillance only					
	*N* = 14		*N* = 16		*N* = 51	
	*N*	%	*N*	%	*N*	%
Age group						
<2 years	1	7.1%	1	6.3%	30	58.8%
2–4 years	2	14.3%	2	12.5%	9	17.6%
5+ years	11	78.6%	13	81.2%	12	23.5%
Influenza type/subtype						
Influenza A	4	28.6%	5	31.3%	n/a	
Influenza B	1	7.1%	2	12.5%	n/a	
pH1N1	9	64.3%	9	56.3%	n/a	
Autopsy performed						
Yes	10	71.4%	11	68.8%	38	74.5%
If yes, findings used to determine cause of death	10	100%	10	90.9%	27	71.1%
Underlying cause of death						
Influenza (J09–J11)	n/a		16	100%	4	7.8%
Pneumonia (J12–J18)	n/a		0		47	92.2%
Other	14	100%	n/a		n/a	

**Table:** (a)

		Laboratory-confirmed influenza	
		pediatric death surveillance	
		Reported	Missed

P&I coded deaths	Reported	16	51
	Missed	14	*z* = 45

*n*_1_ = 30.

*n*_2_ = 67.

*N* = 126 deaths.

**Table:** (b)

		Laboratory-confirmed influenza	
		pediatric death surveillance	
		Reported	Missed

Influenza only coded deaths	Reported	16	4
	Missed	14	*z* = 4

*n*_1_ = 30.

*n*_2_ = 20.

*N* = 38 deaths.
